# Analysis of risk factors associated with surgical treatment for children with foreign body ingestion

**DOI:** 10.3389/fped.2026.1851738

**Published:** 2026-07-27

**Authors:** Huai-Jing Yu, Tian-Yi Zhang, Hong-Chao Yang, Jia-Guo Zhang, Hui-Hui Zhang, Chuan-Tao Ren, Cheng-Fei Li

**Affiliations:** Department of Pediatric Surgery, Qilu Hospital of Shandong University Dezhou Hospital, Dezhou, China

**Keywords:** child, endoscopy, gastrointestinal foreign bodies, magnet, surgery

## Abstract

**Objectives:**

Foreign body ingestion (FBI) is a common pediatric emergency that can lead to significant morbidity. The clinical outcome is significantly influenced by the type, size, and location of the object, as well as how long it has been inside the body for. This study aims to summarize the clinical characteristics of FBI in the digestive tracts of children and analyze the diagnosis, treatment, and risk factors for surgical intervention of digestive tract foreign bodies.

**Methods:**

This retrospective cross-sectional study included all pediatric patients (aged 0–18 years) diagnosed with digestive tract foreign bodies at the Qilu Hospital of Shandong University Dezhou hospital from December 2021 to December 2025. The data included demographic information, clinical manifestations, type, quantity, and location of the FBI, time of ingestion, treatment methods, and complications. The collected data were statistically analyzed using descriptive statistics, chi-square tests, and logistic regression analysis to identify the risk factors for surgical intervention for FBI.

**Outcome:**

A total of 194 children were included in this study. Among them, 71 cases had foreign bodies removed through endoscopy or surgery, while 123 expelled the foreign bodies spontaneously. The male to female ratio was 127:67. The number of children under 3 years old was the largest, with a total of 106 cases (54.64%). Retention time of FBI for less than 24 h occurred in 153 cases (78.87%), 84 cases (43.30%) had symptoms, and FBI being mostly located in the upper digestive tract accounted for 140 cases (72.16%). The foreign bodies were mostly coins and plastics, with 53 cases (27.32%) and 31 cases (15.98%), respectively. There were 76 cases (39.18%) of sharp or corrosive FBI. Foreign body quantity was less than 2 in 168 cases (86.60%), and 32 cases (16.49%) had injuries in the digestive system or other systems. This study divided the 71 cases that had the foreign bodies removed through endoscopy or surgery into the surgical treatment group (11 cases) and the endoscopic treatment group (60 cases). It was found that factors such as age, foreign body type, foreign body quantity, retention time of FBI, the hospitalization time, and bodily injury were significantly related to whether surgical treatment was performed (*P* < 0.05).

**Conclusion:**

Surgical treatment is a method for addressing FBI. Age, foreign body type, foreign bodies quantity, retention time of FBI, and digestive system or other system injury are all risk factors for surgical treatment. This study systematically summarizes the indications and methods of surgical treatment for FBI to provide a reference for clinical decision-making.

## 1 Introduction

Foreign body ingestion (FBI) is a common occurrence in pediatric surgery, and most cases occur in children aged 1–5 years old (1). It is less common in teenagers, but some adolescent patients may exhibit the behavior of swallowing foreign objects due to psychological issues (2). Most of the children have no obvious symptoms. The parents take their children to the hospital due to suspected ingestion of foreign objects. Some of the children may experience abdominal pain and vomiting (3). Of the cases, 80%-90% can be naturally expelled from the body through the digestive tract. 10%-20% of the foreign objects need to be removed through endoscopy. Less than 1% of the children require surgical treatment (4, 5). Surgical treatment, as a method for dealing with digestive tract foreign bodies, aims to address complications caused by digestive tract foreign bodies, such as relieving intestinal obstruction in children and repairing intestinal perforations(6).

Since most patients are unable to provide accurate and effective information about their FBI history, it often leads to delayed diagnosis or even misdiagnosis of the condition (7). Surgical intervention, as a crucial treatment method, mainly addresses complications that cannot be managed through conservative treatment or endoscopic procedures (8, 9). In terms of surgical procedure selection, the principles of damage control and minimally invasive surgery are followed (10). Laparoscopic surgery has become the preferred method for exploration, foreign body removal, and local intestinal resection and anastomosis; for cases with severe abdominal infection, shock, or complex conditions, traditional open surgery is still indispensable for thorough exploration, thorough debridement, and complex reconstruction (8).

This study aims to analyze the risk factors for surgical treatment of children with FBI. Through a retrospective analysis of children with FBI admitted to the pediatric surgery department of Qilu Hospital of Shandong University Dezhou hospital in recent years, this study describes and analyzes the symptoms, treatment methods, and clinical outcomes of these children, as well as the risk factors for complications in these children. The goal is to help pediatricians and pediatric surgeons in handling children with FBI, improve the accuracy of clinical diagnosis, understand the surgical timing, avoid delaying the condition, and prevent the occurrence of serious complications.

## 2 Materials and methods

### 2.1 Study design and participants

This retrospective study was conducted in Qilu Hospital of Shandong University Dezhou hospital. The study collected the electronic medical records of 194 children aged 0–18 years who were suspected or diagnosed with FBI during the period from December 2021 to December 2025. Among them, 118 patients expelled the foreign body through conservative treatment, and five patients had no foreign body found during endoscopic examination and then expelled it on their own. The foreign body was successfully removed from a total of 71 children via endoscopy or surgery. These 71 children were divided into the surgical treatment group and the endoscopic treatment group. The surgical treatment group consisted of those with foreign bodies that were ultimately removed through surgery, and the endoscopic treatment group consisted of those where the foreign body was ultimately removed through endoscopy. Inclusion criteria for cases included 1) a history of swallowing foreign bodies into the digestive tract, 2) those with foreign bodies visible in their stools, 3) those with confirmed digestive tract foreign bodies through endoscopic examination or surgery, 4) those aged between 0 and 18 years; and 5) those with complete clinical data. Criteria for excluding cases included 1) those without foreign bodies detected upon examination, and 2) those with incomplete clinical data.

### 2.2 Data collection

General patient information, including gender, age, family address (urban or rural), retention time of the foreign body, foreign body type, foreign body location, and foreign body quantity, were collected. For the classification of foreign bodies, “other bodies” was used for less common types. Among the patients who underwent surgery, the position of the foreign body was defined as the location identified during the surgery. In patients who did not undergo surgery, the position of the foreign body was defined as the location determined through imaging assessment at the time of admission. “Digestive system or other system injury” referred to any injury arising from the foreign body or its removal.

### 2.3 Inspection method

x-rays were used to detect high-density foreign bodies such as coins, button batteries, and bone fragments. Ultrasound was used for larger foreign bodies or certain non-metallic foreign bodies. A CT scan was used if the x-ray failed to provide a definitive diagnosis or when complications were highly suspected. Endoscopy or surgical treatments were performed if the patient had a history of foreign body swallowing and had definite symptoms (such as abdominal pain or cessation of defecation.).

### 2.4 Indication and contraindication for endoscopic and surgical treatment

Indication for endoscopic treatment involved cases of difficult natural discharge of the foreign body in the upper gastrointestinal tract. Contraindication occurred when the foreign body penetrated the digestive tract, when the foreign body was longer than 20 cm in length, or in cases of severe coagulopathy. Surgical treatment occurred when the foreign body could not pass spontaneously and endoscopic treatment failed, with no absolute contraindications.

### 2.5 Statistical analysis

Statistical analysis was conducted using SPSS 25.0 software. Normally distributed measurement data were expressed as (±s), and the independent sample t-test was used for comparison between the two groups; non-normally distributed measurement data were expressed as M (P25, P75), and the rank sum test was used for comparison between the two groups. Count data were expressed as [cases (%)], and the *χ*^2^ test or Fisher's exact probability method was used for comparison between the two groups. Variables with *P* < 0.05 in the univariate analysis were included in the multivariate Logistic regression to analyze the independent risk factors affecting the occurrence of digestive tract foreign body complications. *P* value <0.05 was considered statistically significant.

### 2.6 Follow-up and ethics

At discharge, family members were instructed to seek a re-evaluation at the outpatient clinic or return to the hospital as per the instructions; hospitalization was arranged when there were clinical indications. This study was approved by the Institutional Review Board. Due to the retrospective design, informed consent was waived.

## 3 Results

### 3.1 Demographic information

194 patients were divided into three groups: conservative treatment (*N* = 118), endoscopic treatment (*N* = 65), and surgical treatment (*N* = 11). Of the patients, males accounted for 127 (65.46%) and females for 67 (34.54%). There were 106 cases (54.64%) under the age of 3, 39 cases (20.10%) aged 3–6, 25 cases (12.89%) aged 7–9, and 24 cases (12.37%) aged 10 or above. 153 cases (78.87%) had an ingested foreign body retention time of less than 24 h, 41 cases (21.13%) had a retention time of 24 h or more, 84 cases (43.30%) had symptoms, and 110 cases (56.70%) had no symptoms. 140 cases (72.16%) had the foreign body located in the upper digestive tract, and 54 cases (27.84%) had it located in the lower digestive tract. Foreign bodies that were sharp or corrosive occurred in 76 patients (39.18%). 26 patients (13.40%) had more than two foreign bodies in their digestive tracts. Digestive system or other system injuries were absent in 32 patients (16.49%). There were 132 cases (68.04%) of children whose permanent residence was in urban areas, and 62 cases (31.96%) whose permanent residence was in rural areas (Table 1).

**Table 1:** General information and clinical characteristics of 194 children with FBI.

Project	Total [*n* (%)]	types of treatment		
		Conservative treatment *N* = 118	Endoscopic treatment *N* = 65	Surgical treatment *N* = 11
Sex				
Male	127 (65.46%)	76	46	5
Female	67 (34.54%)	42	19	6
Age				
≤3	106 (54.64%)	65	40	1
3∼6	39 (20.10%)	25	13	1
7∼9	25 (12.89%)	15	8	2
≥10	24 (12.37%)	13	4	7
Retention time of FBI				
<24h	153 (78.87%)	95	57	1
≥24h	41 (21.13%)	23	8	10
Symptoms				
Present	84 (43.30%)	39	36	9
None	110 (56.70%)	79	29	2
Foreign body location				
Upper digestive tract	140 (72.16%)	79	60	1
Lower digestive tract	54 (27.84%)	39	5	10
Foreign body type				
Coin	53 (27.32%)	33	20	0
Battery	12 (6.19%)	2	9	1
Buckyball	7 (3.60%)	3	1	3
Plastic	31 (15.98%)	21	10	0
Food	19 (9.80%)	8	7	4
Hair	2 (1.03%)	0	0	2
Others	70 (36.08%)	51	18	1
Foreign body nature				
Sharp or corrosive	76 (39.18%)	46	23	7
Not sharp or corrosive	118 (60.82%)	72	42	4
Foreign body quantity				
<2	168 (86.60%)	108	58	2
≥2	26 (13.40%)	10	7	9
Digestive system or other system injury				
Present	32 (16.49%)	8	16	8
None	162 (83.51%)	110	49	3
Habitual residence				
Urban	132 (68.04%)	87	40	5
Rural	62 (31.96%)	31	25	6

**Figure 1:**
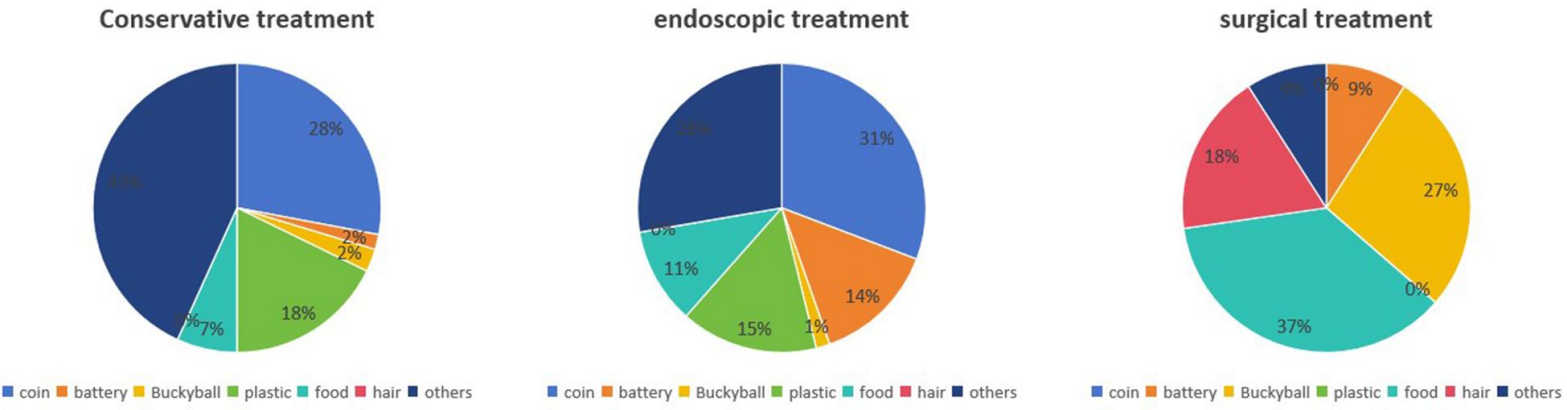
Distribution of three treatments of FBI.

Among the 118 children in the Conservative treatment, 79 cases (66.95%) had no symptoms, 39 cases (33.05%) only showed mild abdominal discomfort, and no signs of peritonitis or intestinal obstruction were observed in the group. The foreign bodies were mostly retained in the upper digestive tract (*N* = 79, 66.95%), mainly consisting of coins (*N* = 33, 27.97%) and plastic products (*N* = 21, 17.80%), and most of them were not sharp or corrosive (*N* = 72, 61.01%). The majority of the cases (*N* = 108, 91.5%) had a single foreign body, and most of the children did not have digestive system or other system injuries (*N* = 110, 93.22%). The distribution of foreign bodies in the conservative treatment group, endoscopic treatment group, and surgical treatment group is shown in Figure 1.

### 3.2 Flowchart of patient inclusion in the research process

**Figure 2:**
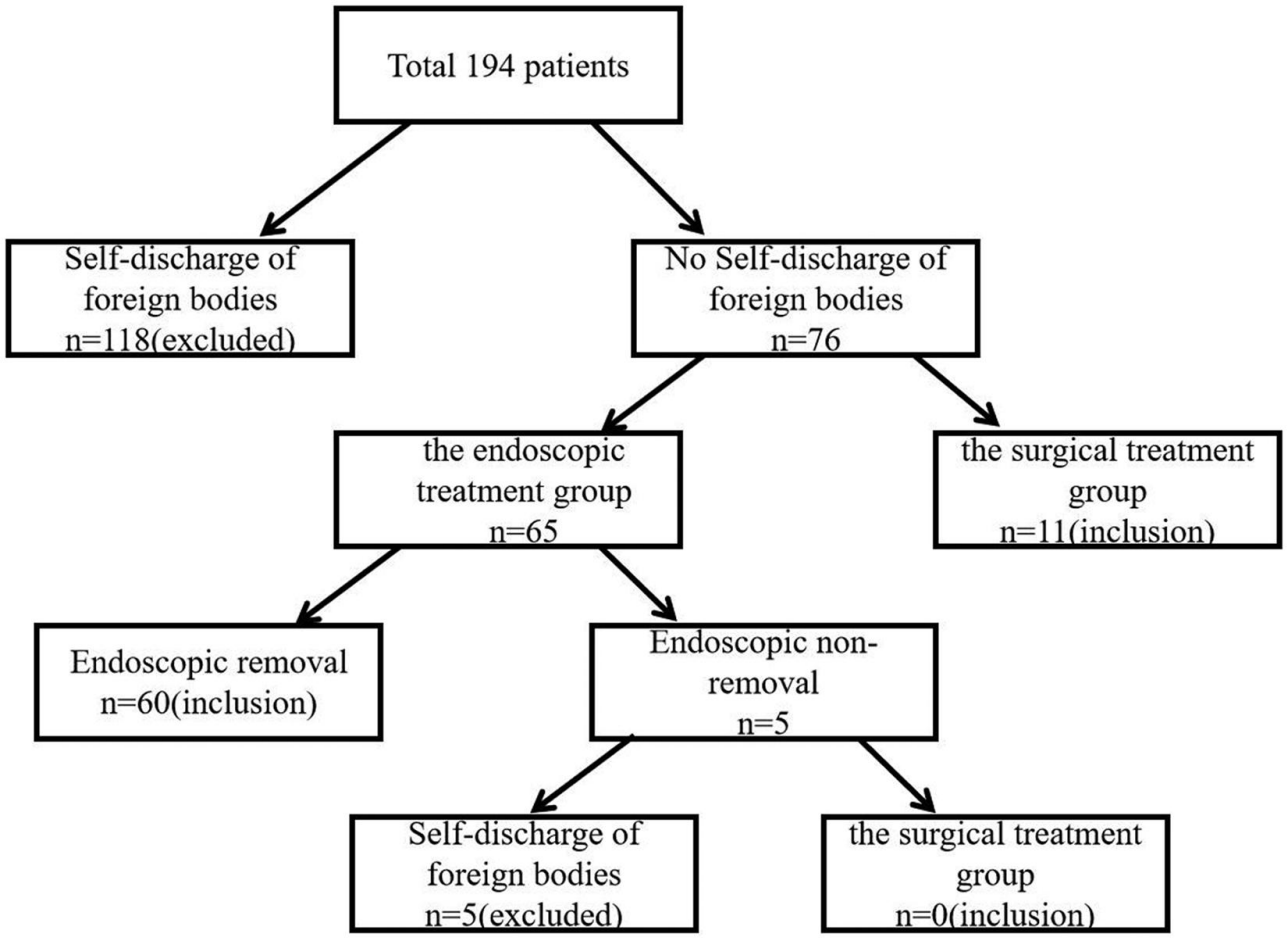
Flowchart of patient inclusion and exclusion.

During the study period, 194 patients presented with confirmed FBI. Exclusions were as follows: 118 with FBI self-discharged the foreign bodies, 5 with FBI did not have the foreign body found after endoscopic examination but then self-discharged. The final analytic cohort consisted of 71 patients (Figure 2).

### 3.3 Analysis of risk factors related to surgical management of FBI

The proportion of older children in the surgical group was significantly higher than in the endoscopy group [11 (7, 11.4) vs. 3 (1.6, 5); *p* < 0.001]. With respect to FBI type, the surgical treatment group had higher proportions of buckyball, food, and hair (*p* < 0.001). Foreign body quantity in the surgical treatment group was significantly higher than that in the endoscopic treatment group (*p* < 0.001). The retention time of foreign bodies in the surgical treatment group was significantly longer than that in the endoscopic treatment group [96 (48.0, 240.0) vs. 3 (2.0, 11.5); *p* < 0.001]. The hospitalization time of the surgical treatment group was significantly longer than the endoscopic treatment group [6 (3.0, 16.0) vs. 1 (1.0, 1.0); *p* < 0.001]. The number of patients with digestive tract or other system injuries in the surgical treatment group was significantly higher than that in the endoscopic treatment group (*P* = 0.005) (Table 2).

**Table 2:** Univariate analysis of risk factors related to surgery treatment of foreign bodies ingestion.

Project	The surgical treatment group	The endoscopic treatment group	*P* Value
Symptoms			
Present	9	34	0.181
None	2	26	
Age(P25.P75)	11 (7.0, 11.4)	3 (1.6, 5.0)	0.001
Sex			
Male	5	42	0.165
Female	6	18	
Foreign body type			
Coin	0	20	<0.001
Battery	1	8	
Buckyball	3	1	
Plastic	0	8	
Food	4	6	
Hair	2	0	
Others	1	17	
Foreign body quantity			
<2	2	54	<0.001
≥2	9	6	
Foreign body nature(sharp or corrosive)			
Present	7	21	0.098
None	4	39	
Retention time of FBI (P25. P75)	96 (48.0, 24.0)	3 (2.0, 11.5)	<0.001
The hospitalization time	6 (3.0, 16.0)	1 (1.0, 1.0)	<0.001
Digestive system or other system injury			
Present	8	16	0.005
None	3	44	
Habitual residence			
Urban	5	37	0.249
Rural	6	23	

To explore the risk factors for surgical treatment of FBI, the influencing factors that showed statistically significant differences in the above single-factor analysis were included in the multivariate logistic regression model. Age, foreign body type, foreign body quantity, retention time, hospitalization time, and digestive system or other system injury were risk factors for complications of digestive tract foreign bodies (*P* < 0.05) (Table 3).

**Table 3:** Multivariate analysis of risk factors related to complications of foreign bodies ingestion.

Factor	Adjusted odd ratio	95% CI	*P* Value
Age	1.723	1.416–2.098	<0.001
Foreign body type			
Battery	2.13	0.41–11.13	0.372
Buckyball	51	11.236–231.496	<0.001
Food	11.33	4.39–29.28	<0.001
Foreign body quantity	41	17.607–95.476	<0.001
Retention time of FBI	1.027	1.016–1.037	<0.001
The hospitalization time	2.076	1.675–2.573	<0.001
Digestive system or other system injury	7.767	3.862–15.621	<0.001

### 3.4 A case of perforation of the ileum caused by multiple magnetic FBI of a child due to delayed medical treatment

**Figure 3:**
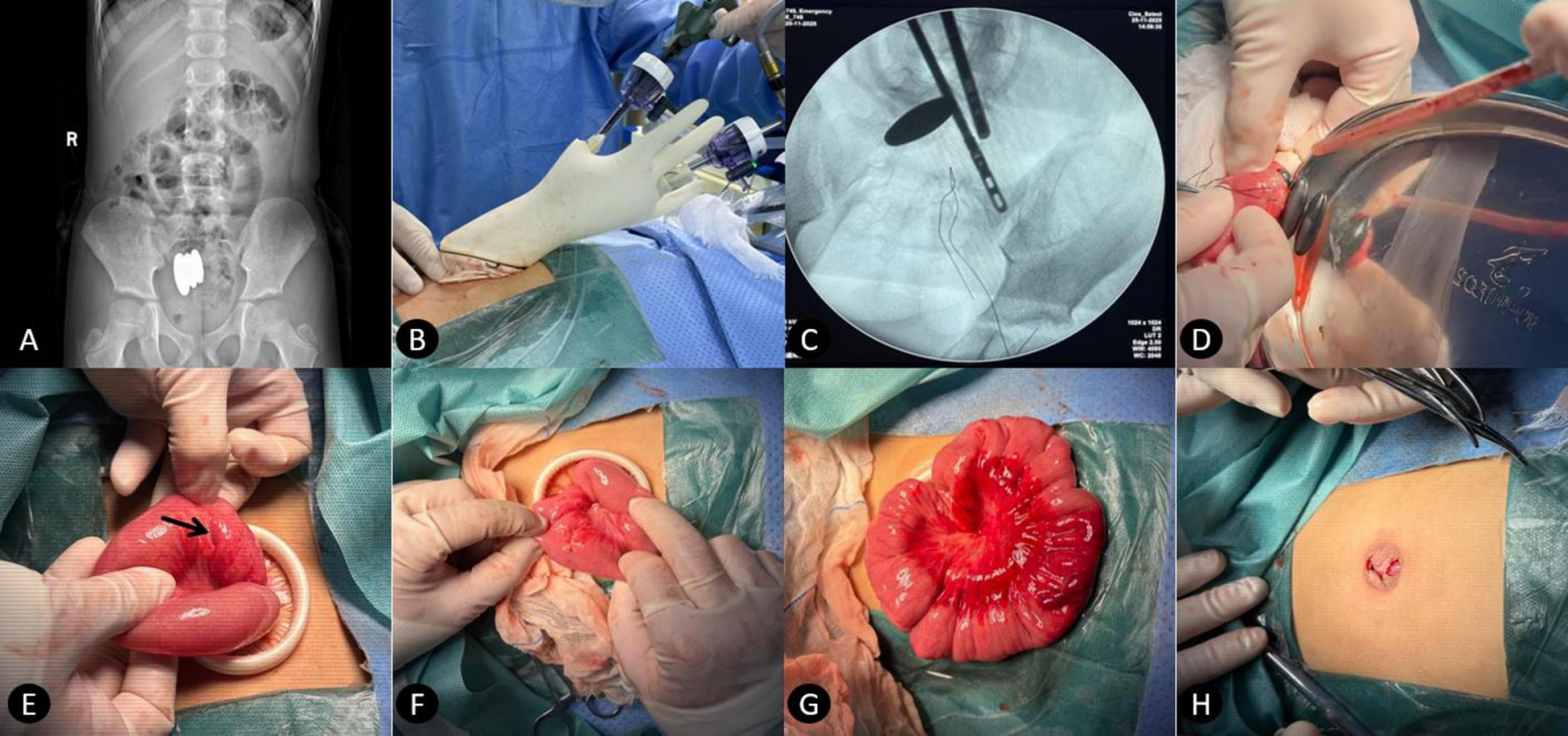
Images of patients in the surgical treatment group during the operation. **(A)** x-ray images show three Buckyballs at the lower end of the small intestine; **(B)** Sterile gloves should be placed over the barrier retractor and the finger part of the sterile gloves opened, the trocar entered, and the part connecting the gloves and the trocar tightened to increase the operating space of the laparoscope; **(C)** During the operation, the C-arm x-ray machine is used to explore the location of the foreign body; **(D)** All three foreign bodies were completely removed; **(E)** At the magnetic suction site, a clear perforation can be seen in the intestine (the arrow points to the perforation site); **(F,G)** Postoperative intestinal manifestations; **(H)** During the operation, Z-plasty was used to expand the incision while ensuring the aesthetic appearance of the wound.

Most FBI patients visited the hospital within 0.5 hours to 10 days. Children with FBI retention often had obvious abdominal symptoms and digestive system damage (5). In this study, a 7-year-old child was admitted to the hospital due to “swallowing 3 Buckyballs for 4 days, abdominal pain accompanied by vomiting for 1 day (Figure 3). The child experienced abdominal pain, which was intermittent, and was accompanied by vomiting. The vomitus was gastric contents. Subsequently, an X-ray examination was performed at our hospital (Figure 3A), revealing three Buckyballs, two measuring 30*10 mm and one measuring 43*15 mm. Physical examination: the abdomen was soft, with deep tenderness in the left lower abdomen, and a hard FBI could be felt upon deep palpation. Considering that the child had an excessively long retention time of FBI and obvious abdominal symptoms, a surgical exploration was decided upon. The abdominal cavity was entered for exploration, and the two Buckyballs were found at the Treitz ligament. Further exploration revealed no remaining Buckyballs. Subsequently, combined with C-arm X-ray machine and laparoscopy, exploration was continued (Figures 3B, C). At a distance of approximately 10 cm from the ileocecal region, there was a layer of white pus on the ileal wall tissue, accompanied by perforation (Figure 3E). There was one Buckyball beside the perforation. Three FBI were removed from the perforation site (Figure 3D), the small intestinal perforation was repaired (Figures 3F, G), and the intestinal tract was explored, with no abnormalities found. The abdomen was closed (Figure 3H).

## 4 Discussion

FBI in children is mainly related to their strong curiosity and habit of exploring new things with their mouths. As children's toys are constantly being updated and improved, such as Lego sets and electronic products with button batteries, the risk of FBI significantly increases. Some countries have imposed restrictions on the use of magnets in toys. For example, in the UK, magnetic toys are required to have warning labels and, in New Zealand, such products are prohibited from being sold (11). Therefore, it is necessary to strengthen children's education and family supervision to prevent FBI. This study included 194 children with FBI. Among them, 118 cases were discharged spontaneously after conservative treatment, 65 cases received endoscopic treatment, and 11 cases underwent surgical treatment. Among the 65 cases treated with endoscopy, five cases had no foreign body seen under the endoscope and later discharged the item spontaneously after short-term observation. For those with a clear medical history and no symptoms but negative endoscopic results, repeated endoscopic operations should be avoided. Most foreign bodies can be expelled spontaneously. Although endoscopic treatment offers the advantages of less trauma and faster recovery, for multiple foreign bodies, longer retention time, or complete intestinal obstruction, endoscopy treatment may delay the condition and surgical treatment should be chosen.

This study found that foreign body being harmful (sharp or corrosive) had no significant statistical association with whether surgical treatment was ultimately performed. This phenomenon is likely due to the fact that clinicians have developed a highly unified emergency treatment consensus for high-risk foreign bodies (12, 13). Take button batteries as an example—their harm is far more than simple mechanical compression (14). It also involves tissue burns caused by short-circuiting and heat release, chemical corrosion caused by the leakage of electrolytes, and protein reactions with the mucosa. The combined effect of multiple mechanisms can lead to mucous membrane liquefaction and necrosis, perforation, and even mediastinal infection within a short period of time. Take button batteries as an example—their harm is far more than simple mechanical compression (15). Therefore, for such clearly high-risk foreign bodies, the clinical standard is that, regardless of where they are located in the digestive tract, immediate endoscopic removal is required (16). When evaluating the predictive factors for surgical treatment, whether the emergency endoscopic intervention for high-risk foreign bodies has been strictly implemented may be a more crucial confounding variable or prerequisite than the properties of the foreign bodies.

FBI was particularly prevalent among toddlers (17). The highest incidence occurs in ages 4.5–5, and is more common in boys (12). However, in this study, the proportion of elder children in the surgical group was significantly higher. Most of the children were in the adolescent stage (10–13 years old). During follow-up of the surgical treatment group, it was revealed that one 7-year-old child had swallowed three objects after accepting a bet with classmates, without informing his parents. This eventually led to intestinal perforation. An emergency surgery was performed to remove the foreign objects and repair the intestinal perforation. Another child suffered from obsessive-compulsive disorder and depression and had been swallowing hair for a long time, causing the hair to clump together and resulting in intestinal obstruction. The patient underwent surgical treatment to remove the foreign objects. Previous studies have identified patients with mental illnesses, such as intellectual disability/neurodevelopmental delay, depression, and autism, are at increased risk for FBI (18, 19). Although there is a lack of large-scale studies specifically targeting FBI patients with mental illnesses, this factor should be regarded as high-risk and requires further research. Furthermore, the caregivers and educators of these children should pay attention to such incidents and provide targeted supervision (20).

For FBI, there have been few studies on surgical intervention.. Previous studies mostly focused on the related risk factors of endoscopic intervention (7, 17). This study specifically analyzed the risk factors of surgical intervention. For children with FBI, surgery is not the preferred option (4). However, when FBI causes complications or when conservative treatment and endoscopic treatment fail, timely surgical intervention is the key to saving lives (21). The surgical indications are mainly based on the child's symptoms, the duration of foreign body retention, the type of complications, the characteristics of FBI, and the failure of conservative and endoscopic treatments (6). In the treatment of FBI, both endoscopic and surgical treatments have their own advantages and disadvantages. The advantages of endoscopic treatment are that it is minimally invasive, offers a rapid recovery, is low cost, and is suitable for FBI in the esophagus to duodenum and colon with suitable morphology and no signs of perforation (22). However, it cannot reach the small intestine, it is difficult to deal with multiple magnetic foreign bodies that have been impacted by perforation or attracted across intestinal segments, and may aggravate the injury during operation. Surgical treatment covers the whole digestive tract, can repair perforations, offers resection of the necrotic intestinal segment, provides thorough debridement, is a definitive plan for endoscopic failure, and can address small intestinal foreign bodies, complete intestinal obstruction, and peritonitis. But it is more traumatic, requires more anesthesia, has a long recovery time, and costs more. In clinical practice, endoscopic treatment is given priority for suitable foreign bodies in the upper digestive tract. In the event of perforation, strangulation obstruction, retention of the small intestine, or attraction of multiple magnetic beads, surgery was performed directly. The two methods are not in opposition but individualized according to the location of the foreign body, complications, and technical conditions. If necessary, the combination of the two endoscopic methods can be used.

When deciding on surgical intervention for children with FBI, the principle of individualized treatment based on evidence-based medicine must be followed (13). To ensure the treatment is both safe and appropriate, minimally invasive techniques should be applied (9). Laparoscopic surgery is the preferred surgical method, which is mostly applicable when the location of the foreign body is relatively clear and there is no diffuse peritonitis or extensive abdominal adhesions (22). When dealing with intestinal wall compression or localized perforation caused by magnetic foreign bodies, laparoscopy can locate the position of the foreign body, then the intestinal tube can be removed out of the body and operations such as intestinal incision and FBI removal, local intestinal segment resection, and anastomosis can be performed (23). When children develop infectious shock, or if diffuse suppurative peritonitis, multiple intestinal perforations, dense adhesions, or a need for systemic exploration of the entire intestinal system are detected during the operation, it is necessary to promptly switch to or directly adopt traditional open abdominal surgery (24). Open abdominal surgery can provide sufficient surgical field exposure, facilitating the surgical operation (25).

During the process of removing foreign bodies, the more challenging aspect is locating the objects. Although x-rays can detect all metallic objects and 86% of glass objects, the sensitivity for detecting fish bones is only 26% (12). During the operation, due to intestinal peristalsis, the position of the foreign object may change, making it difficult to locate the object. For metallic or magnetic foreign objects, endoscopy-assisted foreign body removal can be used to assist in location (26). Nijhawan (27) reported that ferromagnetic coins were removed in a meantime of 60 s (range 30–90 s) and non-ferromagnetic coins were removed in a mean time of 150 s (range 90–180 s). Liu (20) proposed using cylindrical magnetic localization technology during operations to accurately identify and successfully remove all foreign bodies while minimizing tissue trauma. Tomaz (28) used handheld metal detectors during the operation to locate the foreign bodies. They found that the handheld metal detectors had a detection sensitivity of 89.7% and a specificity of 100% for the presence of FBI. In this study, a modified incision establishment and protection technique was used to optimize laparoscopic operation field and instrument flexibility. A “Z”-shaped small incision was made around the umbilicus. Combined with the use of barrier retractor and sterile gloves, a sealed and expandable operating channel was constructed. This method effectively expanded the range of movement of the abdominal puncture point while maintaining the stability of the pneumoperitoneum. It created a more sufficient and flexible operating space for systematic exploration of the abdominal cavity, localization of foreign bodies, and safe removal, thereby enhancing the safety and efficiency of the surgery.

This is a retrospective study of a single center, lacking the experience from other centers. The overall sample size in this study, particularly the number of children in the surgical treatment group, was limited, which may affect the accuracy of the statistical analysis. Due to the differences in living habits in other regions, it may lead to different clinical characteristics of FBI in various regions. Additionally, due to the different choices of departments for treatment by parents, cases of FBI in other departments such as otorhinolaryngology and digestive internal medicine may be missed.

## 5 Conclusion

In conclusion, FBI in children is a common surgical emergency. The advantages of surgical treatment lie in identifying intestinal perforation, peritonitis, and refractory obstructions that cannot be handled by endoscopy. In terms of treatment methods, minimally invasive techniques such as laparoscopy are widely used due to their advantages of small trauma and quick recovery. However, traditional open surgery is still indispensable in handling complex and critical cases. The final surgical approach should follow the principle of individualization, aiming to relieve obstruction, repair damage, control infection, and save lives. For clinicians, prevention is better than treatment. It is necessary to educate parents on keeping their children from handling small bodies that are prone to being swallowed and to cultivate good eating habits. If a child accidentally swallows a foreign body, they should seek medical attention promptly.

## Data Availability

The original contributions presented in the study are included in the article/Supplementary Material; further inquiries can be directed to the corresponding author.
